# The impact of the relationship between lesion diameter and total core length on the detection rate of clinically significant prostate cancer for PI-RADS 3 lesions

**DOI:** 10.1007/s00345-024-04845-1

**Published:** 2024-03-15

**Authors:** Emrah Yakut

**Affiliations:** Department of Urology, Yuksek Ihtisas University, İşçi Blokları, 1588. Cd. No: 18/A, 06520 Çankaya, Ankara Turkey

**Keywords:** Clinically significant prostate cancer, Lesion diameter, Multiparametric magnetic resonance imaging, Prostate fusion biopsy, Total core length

## Abstract

**Background:**

The aim of our study was to determine the effect of total core length (TCL) for prostate imaging reporting and data system (PI-RADS) 3 lesions to facilitate clinically significant prostate cancer (csPCa) detection based on the lesion diameter.

**Materials and methods:**

A total of 149 patients with at least 1 lesion with a PI-RADS 3 were evaluated retrospectively. The lesions with diameters of < 1 cm were categorized as small lesions and lesions of ≥ 1 cm were categorized as large lesions. The lengths of biopsy cores from PI-RADS 3 lesions were summed for each lesion separately, and TCL was calculated. The relationship between TCL and csPCa was analyzed separately for the small and large groups with multiple logistic regression analyses.

**Results:**

A total of 208 lesions were detected by multiparametric magnetic resonance imaging (MpMRI) in 149 males included in the study. The mean TCL was 44.68 mm (26–92) and the mean lesion diameter was 10.73 mm (4–27) in PIRADS 3 lesions. For small diameter lesions (< 1 cm), the odds of finding clinically insignificant prostate cancer (ciPCa) increase by 1.67 times if TCL increases by one unit. Hence, increasing TCL for small lesions only increases the odds of ciPCa detection. For large diameter lesions (≥ 1 cm), if TCL increases by one unit, the odds of finding ciPCa increase 1.13 times and the odds of finding csPCa increases1.16 times. Accordingly, large lesions are more likely to have both csPCa and ciPCa as TCL increases.

**Conclusions:**

Our study showed that for PI-RADS 3 lesions, both more csPCa and more ciPCa were detected as TCL increased. However, in lesions with a size of < 1 cm, only ciPCa was detected more frequently as TCL increased. In conclusion, taking more and longer biopsy cores in PI-RADS 3 lesions below 1 cm does not contribute to the detection of csPCa.

## Introduction

It is aimed to detect clinically significant prostate cancer (csPCa) to optimize follow-up and treatment in patients evaluated with suspected prostate cancer (PCa). Multiparametric magnetic resonance imaging (MpMRI) helps clinicians in this regard. In MpMRI, the prostate gland is mapped and suspicious lesions are found. Specimens are taken from suspicious lesions for MpMRI/transrectal ultrasound fusion-guided prostate biopsy (PFB) [1]. The aim is to improve biopsy yield by targeting suspicious lesions and minimizing the risk of an unnecessary diagnosis of clinically insignificant prostate cancer (ciPCa) [2, 3].

A guideline called ‘Prostate Imaging Reporting and Data System’ (PI-RADS) was developed by the European Society of Urogenital Radiology (ESUR) in 2012 to standardize Mp-MRI evaluation and reporting [4] and updated in 2019 (PI-RADS v2) [5]. The risk of csPCa was classified as ‘very low’ for PI-RADS 1, ‘low’ for PI-RADS 2, ‘high’ for PI-RADS 4, and ‘very high’ for PI-RADS 5. PI-RADS 3 lesions were defined as ‘the presence of clinically significant prostate cancer is uncertain’ [5]. PI-RADS v2 reveals clearly that biopsy should be performed for PI-RADS 4 and 5 lesions, whereas for PI-RADS 3 lesions, it does not provide a clear approach to what should be done [6, 7].

The diameter of suspicious lesions detected on MpMRI may also affect the frequency of csPCa detection. Lesions over 10 mm in diameter have been reported to have a higher frequency of csPCa detection [8, 9]. However, it has also been reported that the greater the length of the biopsy cores from the lesions, the more Pca was captured [10, 11]. Hence, in our study, we investigated the effect of the relationship between lesion diameters and total core lengths (TCL) on the detection rate of csPCa in PI-RADS 3 lesions in which the need for biopsy was not completely clear. The aim of our study was to determine the effect of TCL for PI-RADS 3 lesions to facilitate csPCa detection based on the lesion diameter.

## Materials and methods

### Study design

In this study, we retrospectively analyzed the data of 231 patients who underwent MpMRI-guided PFB in the Urology Department of Yuksek Ihtisas University Memorial Ankara Hospital between 2017 and 2021. Only 149 patients with PI-RADS 3 lesions detected on MpMRI were included in the study. A total of 208 PI-RADS 3 lesions detected in 149 patients were evaluated. Studies have previously demonstrated that lesion sizes are correlated with clinical parameters [12]. This is especially evident in lesions smaller than 1 cm [13]. Having been inspired by these studies, we classified lesions with diameters of < 1 cm as small lesions and lesions of ≥ 1 cm as large lesions. The lengths of biopsy cores from PI-RADS 3 lesions were summed for each lesion separately, and TCL was calculated. The relationship between TCL and csPCa was analyzed separately for the small and large groups. This study was approved by the ethics committee of Omer Halisdemir University (No.20.23.22).

### Data selection

All patients who underwent a biopsy for the first time and those who had previously undergone a biopsy were included in the study. Clinically age, total PSA, prostate volume, radiologically lesion number and lesion diameter, PI-RADS classification [since the study included patients from 2017 to 2021, the lesions on MpMRI were evaluated according to both PI-RADS version 2 and version 2.1 [5, 14]], and the pathologically International Society of Urological Pathology (ISUP) grade group (GG) was used [11, 15]. csPCa was described as ISUP GG ≥ 2. Those with ISUP GG 1 were defined as clinically insignificant prostate cancer (ciPCa). The inclusion criteria were as follows: (i) having undergone 3-T MpMRI, and (ii) having a lesion with a PI-RADS v2 score of 3. Exclusion criteria were (i) no 3-T MpMRI (ii) any contraindication for MRI, and (iii) no PFB results. The study population do not include patients with PI-RADS 4 or 5 concomitant lesions. Systematic biopsy data after PFB were also excluded from the study.

### Multiparametric MRI examination and image analysis

3.0-T Discovery MR750 HDx was used for MpMRI. The imaging protocol included thin-Sect. (3mm) turbo spin echo T2-weighted images in the transverse, sagittal, and coronal planes and dynamic contrast-enhanced images. We performed axial diffusion-weighted imaging with *b* values of 0, 500, 1000, and 1500 s/mm2. All MpMRI results were evaluated by a single radiologist with more than 10 years of experience in prostate MRI. PI-RADS v2 structured scoring criteria developed by the European Society of Urogenital Radiology (ESUR) and the American College of Radiology (ACR) were used to evaluate lesions suspicious for prostate cancer [14]. Among the lesions with PI-RADS 3, those with a diameter of < 1 cm were classified as the small group and those with a diameter of ≥ 1 cm were classified as the large group.

### Biopsy procedure and histopathology

Prostate fusion biopsy was performed by a single urologist under the guidance of a UroNAV device and general electronic ultrasonography (USG). All patients underwent periprostatic nerve blockade with 10 ml lidocaine using a 22-gauge needle. A minimum of 3 and a maximum of 8 biopsies were taken with an 18-gauge biopsy needle from the center of the lesions drawn by synchronizing the MpMRI and USG images. Biopsy core lengths were collected separately for each lesion, and TCL was determined. All the pathology results were evaluated by a single pathologist with more than 10 years of experience in prostate cancer.

### Statistical analysis

In data analysis, descriptive statistical measures (frequencies and percentages) were first presented. Simple and multiple logistic regression analyses were conducted to determine the impact of TCL on csPCa. Moreover, one-way ANOVA was conducted to compare the csPCa and ciPCa groups. SPSS (version 25, SPSS Inc., Chicago, IL, USA) and Microsoft Office Excel software were used for data analysis. A *p* value of less than 0.05 in the 95% confidence interval was considered to indicate statistical significance.

## Results

The socio-demographic characteristics of the patients included in the study are presented in Table 1. PFB was performed for 208 PI-RADS 3 lesions. It was determined at pathology that 95 (63.75%) of the 149 patients were benign, 36 (24.16%) had ciPCa, and 18 (12.08%) had csPCa. Some of the benign and ciPCa lesions were taken from the same patients. Hence, there were 95 benign patients with 144 benign lesions and 36 ciPCa patients with 46 ciPCa lesions. All 18 lesions with csPCa were from separate patients. The effect of TCL on csPCa in biopsies from PI-RADS 3 lesions was tested, and the results are presented in Table 2. For PI-RADS 3, TCL appears to be effective on both csPCa and ciPCa compared with the referenced benign group (*p < *0.05). Accordingly, for PI-RADS 3, if TCL increases by one unit, the odds of finding ciPCa increase by 1.25 times. Similarly, if TCL increases by one unit, the odds of finding csPCa increase by 1.18 times. Thus, the higher the TCL for PI-RADS 3, the higher the odds of diagnosing both ciPCa and csPCa.

**Table 1:** Values on socio-demographic characteristics of the patients

Variables	Variable levels	*f*	%
Number of lesions	1	97	65.1
	2	48	32.2
	3	5	2.7
Type of anesthesia	Local	92	61.7
	General	57	38.3
Pathology	Benign	95	63.75
	ciPCa	36	24.16
	csPCa	18	12.08
Total		149	100

	*N*	Min–max		SD
Continuous variables	Continuous variables	Continuous variables	Continuous variables	Continuous variables
Age (years)	149	44–87	60.81	7.36
PSA (ng/ml)	149	1–33	7.62	4.55
PV (ml)	149	12–140	55.72	26.11
TCL (mm)	208	26–92	44.68	13.29
Lesion diameter (mm)	208	4–27	10.73	3.19

*ciPC* clinically insignificant prostate cancer, *csPC* clinically significant prostate cancer, *PSA* prostate specific antigen, *PV* prostate volume, *TCL* total core length

**Table 2:** Multi-category logistic regression analysis of the effect of PI-RADS 3 TCL on csPCa

Diagnosis (dependent variable)	*B*	Wald	*p*	Exp(*B*)
ciPCa				
Constant	− 11.71	59.34	0.000	
TCL	0.22	54.05	0.000	1.25
csPCa				
Constant	− 13.75	45.97	0.000	
TCL	0.24	41.54	0.000	1.18

Model fit information		Cox & Snell *R*^2^	Nagelkerke *R*^2^	Model data fit (Goodness of fit)	
Chi-square (sd)	152.67(2)	0.52	0.65	Chi-square (sd)	69.93 (92)
*p*	0.000			*p*	0.958
Correct classification percentage (%)					83.2

After the analysis was performed on all lesions, the lesions were divided into two groups, 91 small (0–9 mm in diameter) and 117 large (10 mm and above), according to the lesion diameters, and the logistic regression analysis was repeated. When Table 3 is analyzed, it is noticed that small lesions have an impact on ciPCa compared to the benign group with TCL as reference (*p < *0.05). When Exp (*B*) values are analyzed, for PI-RADS 3 small diameter lesions, the odds of finding ciPCa increase by 1.67 times if TCL increases by one unit. Hence, increasing TCL for small lesions only increases the odds of ciPCa detection. When Table 4 is examined, it is seen that large lesions have an impact on both csPCa and ciPCa compared to the TCL-referenced benign group (*p < *0.05). When Exp (*B*) values are analyzed, for PI-RADS 3 large diameter lesions, if TCL increases by one unit, the odds of finding ciPCa increase 1.13 times and the odds of finding csPCa increases 1.16 times. Accordingly, large lesions are more likely to have both csPCa and ciPCa as TCL increases.

**Table 3:** Multi-category logistic regression analysis of the effect of PI-RADS 3 small-diameter lesions (0–9 mm in diameter) on csPCa

Diagnosis (dependent variable)	*B*	Wald	*p*	Exp(*B*)
ciPCa				
Constant	− 23.96	7.20	0.007	
TCL	0.51	6.33	0.012	1.67
csPCa				
Constant	− 8.23	1.04	0.309	
TCL	0.11	0.27	0.603	1.11

Model fit information		Cox & Snell *R*^2^	Nagelkerke *R*^2^	Model data fit (Goodness of fit)	
Chi-square (sd)	82.64 (2)	0.60	0.84	Chi-square (sd)	15.17(62)
*p*	0.000			*p*	1.000
Correct classification percentage (%)					93.4

**Table 4:** Multi-category logistic regression analysis of the effect of PI-RADS 3 large-diameter lesions (10 mm in diameter and larger) on csPCa

Diagnosis (dependent variable)	*B*	Wald	*p*	Exp(*B*)
ciPCa				
Constant	− 10.60	29.62	0.000	
TCL	0.19	26.89	0.000	1.13
csPCa				
Constant	− 13.30	29.13	0.000	
TCL	0.23	27.31	0.000	1.16

Model fit information		Cox & Snell *R*^2^	Nagelkerke *R*^2^	Model data fit (Goodness of fit)	
Chi-square (sd)	80.48(2)	0.50	0.60	Chi-square (sd)	74.61(82)
*p*	0.000			*p*	0.707
Correct Classification Percentage (%)					77.8

We divided the patients into two groups: those who had a biopsy for the first time and those who had a biopsy previously, and we analyzed the effect of TCL on csPca in both groups. As can be seen in Table 5, TCL of the patients who had their first biopsy has a significant effect on both ciPCA and csPCa in comparison to the benign reference group (*p < *0.05). Moreover, when Exp (*B*) values are analyzed, it is seen that both values are very close to each other. Thus, if TCL increases by one unit, ciPCa probability increases 1.25 times, and csPCa probability increases 1.28 times. Besides, when Table 6 is examined, it is seen that the TCL of patients who had a previous biopsy is effective on ciPCa (*p < *0.05) but not on csPCa (*p* > 0.05), compared to the reference benign group. In addition to that, Exp (*B*) values suggest a 1.33-fold rise in the odds of finding ciPCa if TCL increases by one unit. This indicates that an increase in TCL increases the probability of detecting csPCa in patients undergoing prostate biopsy for the first time, but does not increase the percentage of csPCa detection in patients with previous biopsy.

**Table 5:** Multi-category logistic regression analysis of the effect of PI-RADS 3 TCL on csPCa (patients who underwent prostate biopsy for the first time)

Diagnosis (dependent variable)	*B*	Wald	*p*	Exp(*B*)
ciPCa				
Constant	− 11.93	47.24	.000	
TCL	0.22	43.23	.000	1.25
csPCa				
Constant	− 14.07	39.20	.000	
TCL	0.24	35.94	.000	1.16

Model fit information		Cox & Snell *R*^2^	Nagelkerke *R*^2^	Model data fit (Goodness of fit)	
Chi-square (sd)	125.53(2)	0.53	0.60	Chi-square (sd)	73.98(90)
*p*	0.000			*p*	0.984
Correct classification percentage (%)					81.8

**Table 6:** Multi-category logistic regression analysis of the effect of PI-RADS 3 TCL on csPCa (patients who underwent prostate biopsy for previously)

Diagnosis (dependent variable)	*B*	Wald	*p*	Exp(*B*)
ciPCa				
Constant	− 14.02	10.75	.001	
TCL	0.28	10.15	.001	1.33
csPCa				
Constant	− 6.93	2.12	.145	
TCL	0.09	0.62	.429	0.87

Model fit information		Cox & Snell *R*^2^	Nagelkerke *R*^2^	Model data fit (Goodness of fit)	
Chi-square (sd)	30.01(2)	0.50	0.71	Chi-square (sd)	36.02(42)
*p*	0.000			*p*	0.730
Correct classification percentage (%)					90.7

*ciPC* clinically insignificant prostate cancer, *csPC* clinically significant prostate cancer, *TCL*, total core length

Reference group: benign

After analyzing the effects of patients’ TCL on the diagnosis, cutoff values were determined based on the group memberships produced by logistic regression in order to determine their critical values in determining the diagnosis. When determining cutoff values, an optimal TCL value to detect csPCa could not be determined for all patients. For smaller lesions, however, a TCL of 54 mm or greater increases the likelihood of detecting ciPCa.

## Discussion

It has been well-documented that MpMRI-guided PFB is the most effective method for csPCa detection [16]. The impact of total biopsy core length according to the lesion diameter on the diagnosis of csPCa in PI-RADS 3 lesions, which is the aim of our study, has not been previously studied. In our study, increasing TCL by taking more biopsies from lesions increased the odds of detection of both csPCa and ciPCa in PI-RADS 3 lesions. When the lesions were divided into small (0–9 mm) and large (10 or more mm) lesions according to their diameter, TCL increased in small lesions, and only ciPCa was more likely to be detected, whereas, in larger lesions, both csPCa and COPC were detected more frequently as TCL increased. For PI-RADS 3 lesions with a diameter of < 10 mm, it was concluded that it may be pointless to increase the number of biopsy cores and reach higher TCL values.

Studies have revealed that MpMRI reduces the frequency of ciPCa detection [17]. The presence of csPCa in PI-RADS 3 lesions is considered suspicious according to PI-RADS v2 guidelines [4]. In the guidelines updated in 2019 (PI-RADS v2.1) [5], there are no changes in the recommendations for PI-RADS 4 and 5 lesions, whereas there are partial changes for PI-RADS 3 lesions [18]. CsPCa was not detected in a study evaluating the results of PFB performed on patients with PI-RADS 3 lesions identified using PI-RADS version 2 [19]. In another study in which lesions were identified according to PI-RADS v2.1, csPCa was found in 14.8% of PI-RADS 3 lesions. Likewise, the detection rate of csPCa in PFB for PI-RADS 3 lesions ranges from 7 to 20% in prospective, large-scale studies published in the literature [16, 20–22]. In our study, csPCa was detected in 12% of the patients, consistent with the literature. The odds of detecting csPCa in PI-RADS 3 category lesions depend on the quality of the MRI, the techniques used to confirm biopsy findings, and the experience of the radiologist and pathologist. The data used in our study consisted of patients evaluated by a radiologist experienced in prostate imaging and a pathologist experienced in prostate cancer. The frequency of csPCa detection that is consistent with the literature may be attributed to this factor. In clinical trials reported in non-biopsied men, the mean csPCa prevalence of PI-RADS 3 was 20%, compared to 33% in men with a previous negative prostate biopsy [23]. Meanwhile, in our study, both ciPCa and csPCa diagnoses increased with increasing TCL in patients who underwent biopsy for the first time, whereas in patients who underwent biopsy previously, only ciPCa was found to increase with increasing TCL.

The diameter of the lesions as seen on the MpMRI may also give an insight into the outcome of the biopsy. Studies have previously demonstrated that lesion sizes are correlated with clinical parameters [12]. This is especially evident in lesions smaller than 1 cm [13]. Over 1 cm, ciPCa is less frequently detected [12]. Since a sphere of 0.5 cubic centimeters (cc) corresponds to 1 cm, which is the standard limit for ciPCa according to Epstein, we used 10 mm to define the threshold lesion size [15]. In their study, Lee et al. found that the size of the lesion detected by mpMRI was an independent predictor of ciPCa [12]. As well, in our study, ciPCa was detected more frequently in patients with lesion sizes below 1 cm. However, the frequency of csPCa detection increases in lesions with a size of > 1 cm, as similar results were found in our study [24]. It has been suggested that tumor aggressiveness increases above 1.5 cm [14]. In another recent study, Pca aggressiveness increased clinically and histopathologically in cases with a lesion size of > 1 cm. Moreover, our study focused only on the odds of csPCa capture, whereas PCa aggressiveness was not investigated.

In studies investigating core lengths for PCa, it has been previously revealed that the greater the core length, the more PCa will be captured [11, 26, 27]. There are also studies suggesting that there is no significant correlation between core lengths and Pca [28, 29]. The European Association of Urology (EAU) Guidelines state the shortest acceptable core length as 1 cm [30]. It has been determined that the optimal core lengths for prostate biopsy procedures are between 11 and 13 mm to obtain a correct pathologic diagnosis [10, 11]. However, TCL was not investigated in these studies. There are no studies evaluating the relationship between TCL and lesion diameter for any particular lesion. In our study, which we conducted to partially overcome this deficiency, it was revealed that increasing the number of biopsies and core length did not contribute to the diagnosis of csPCa, particularly in lesions with a size of < 1 cm.

Our study also has limitations. It is a non-randomized retrospective trial. All data were collected from a single institution. PFB data were used in the study, but systematic biopsy data were not included. Patients who underwent prostate biopsy for the first, second, and third times were included in the study in total. No cost analysis was made for the procedures. Procedure-related complications were excluded from the study. The sample size may not be relatively large enough. TCL has not been previously studied in the literature, and studies with larger participation are needed. Furthermore, the biopsy cores were not evaluated separately. Cores lengths were summed for PI-RADS 3 lesions and the relationship between TCL and csPCa was analyzed. Therefore, information on the relationship between each core and PCa could not be obtained. Lesion diameters were divided into 2 groups as less than 1 cm and 1 cm and above, and no separate evaluation was made for each diameter. Nevertheless, our study, which is the first in the literature, may provide a high level of evidence for the optimal total length of biopsy cores to be taken from PI-RADS 3 lesions detected by MpMRI according to lesion diameters.

## Conclusion

Our study showed that for PI-RADS 3 lesions, both more csPCa and more ciPCa were detected as TCL increased. However, this increase was not parallel for lesions with a size of < 1 cm. In lesions with a size of < 1 cm, only ciPCa was detected more frequently as TCL increased. In conclusion, taking more and longer biopsy cores in PI-RADS 3 lesions below 1 cm does not contribute to the detection of csPCa. Increasing TCL in previously biopsied patients only results in an increase in ciPCa rates. Further prospective multicenter studies with higher participation are needed.

## Data Availability

The data sets generated during and/or analyzed during the current study are available from the corresponding author upon reasonable request.
